# Polymers Comprising Cholesterol: Synthesis, Self-Assembly, and Applications

**DOI:** 10.3390/ma2020636

**Published:** 2009-06-02

**Authors:** Yuxiang Zhou, Victoria A. Briand, Nitin Sharma, Suk-kyun Ahn, Rajeswari M. Kasi

**Affiliations:** 1 Chemistry Department, University of Connecticut, Storrs, CT 06269, USA; 2 Polymer Program, The Institute of Material Sciences, University of Connecticut, Storrs, CT 06269, USA

**Keywords:** cholesterol, polymer, liquid crystalline, self-assembly, biomaterials, optoelectronics

## Abstract

This article reviews the current status of self-assembling liquid crystalline polymers comprising cholesterol. This article will focus on synthesis, structure-property relationships and strategies to direct ordering and packing of meso- and nanostructures of cholesterol polymers in the neat- or melt state and in solution. The applications of these self-assembled structures will be presented.

## 1. Introduction

**Figure 1:**
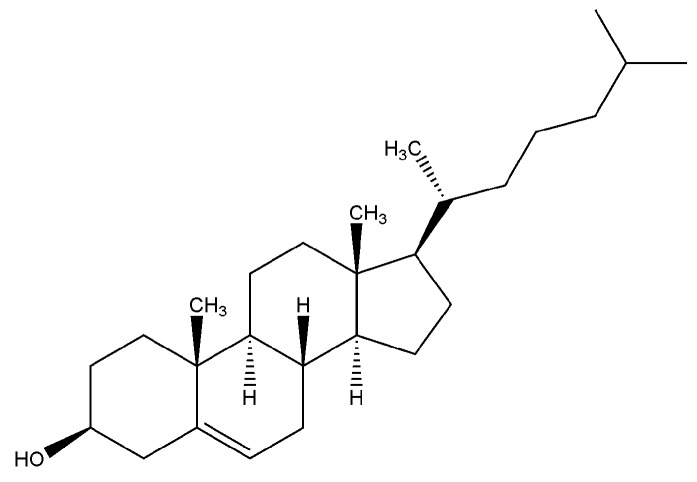
The chemical structure of cholesterol.

Cholesterol (Figure 1) and its derivatives are fat-soluble molecules that occur in nature. Cholesterol derivatives are broadly defined as hydrophobic or amphiphilic, based on the chemical subunits that are present in the molecule. Ordered arrays of cholesterol molecules or mesogens result in the formation of liquid crystalline (LC) mesophases [1,2,3,4,5,6,7,8,9,10,11]. Orientational order arises from parallel arrangement of cholesterol; while, positional order is obtained from attractive forces that hold the assembly together. Mesophase morphology also depends on molecular shape and amphiphilicity of the mesogen such that (1) nematic phases are formed by rod-like or disc-like units that have long range orientational order, (2) cholesteric or helical phases are formed by chiral nematic molecules, and (3) smectic or layered mesophases are formed when rod-like molecules arrange in simple layers (A, B) or as chiral layers (C) [2,3]. Upon heating, these mesophases lose positional and orientational order, eventually resulting in a disordered phase known as the isotropic phase. The lowest temperature at which this disordered phase appears is known as clearing temperature (T_cl_) [1,2,3,4,5,6,7,8,9,10,11].

This review article will focus on the synthesis and structure-property relationships of cholesterol-based polymers. These polymers are synthesized to harness both the mechanical properties of physically or chemically cross-linked polymer networks as well as the unique features of cholesterol such as chirality, amphiphilicity, liquid crystallinity, and biocompatibility. These polymers self-assemble due to various interactions to form a plethora of interesting and exotic LC structures in the neat- and solution states. The applications of these self-assembled structures will also be investigated.

## 2. Synthesis of Polymers Bearing Cholesterol

### 2.1. Main-chain polymers

Main-chain polymers containing cholesterol are linear polymers end-capped with cholesterol. These polymers are synthesized by two methods: (1) cholesterol moieties that initiate polymerization of monomers and (2) cholesterol introduced via post-polymerization reaction between the end-groups of the precursor polymers and cholesterol or cholesteryl chloroformate. Examples of main-chain polymers bearing cholesterol end-groups include poly(*N*-isopropylacrylamide), poly(ethylene glycol), polycarbonate, poly(lactide-co-glycolide), polyisoprene, poly(ε-caprolactone), poly(2-(acrylamido)-2-methylpropanesulfonic acid), and polylactides [12,13,14,15,16,17,18,19,20,21,22,23,24,25,26].

**Figure 2:**
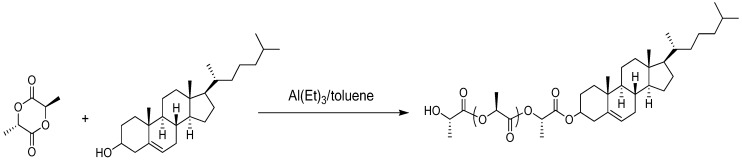
Synthesis of cholesteryl-oligo(L-lactic acid).

An example of method 1 is the synthesis of cholesteryl-oligo(L-lactic acid) (Figure 2). Cholesterol is pre-treated with Al(Et)_3_ in toluene. The resulting aluminum alkoxide is used to ring open and polymerize L-lactide to produce the cholesteryl-oligo(L-lactic acid) [24].

**Figure 3:**
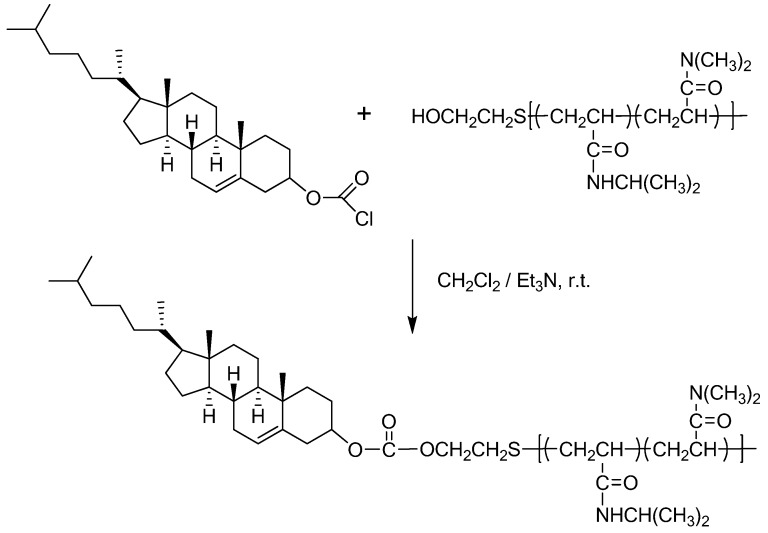
Synthesis of Chol-P(NIPAAm-co-DMAAm).

In method 2, cholesteryl end-capped poly(*N*-isopropylacrylamide-co-*N,N*-dimethylacrylamide), Chol-P(NIPAAm-co-DMAAm), has been prepared (Figure 3). Precursor copolymer P(NIPAAm-co-DMAAm) with a hydroxyl end-group was first prepared by using 2-hydroxylethanethiol as a chain transfer agent. The hydroxyl end-group can undergo esterification reaction with cholesteryl chloroformate, resulting in the product copolymer with cholesterol end-group [26].

The synthesis, self-assembly and applications of these main-chain polymers are summarized in Table 1.

**Table 1:** Types of main-chain homo- and copolymers.

Abbreviation	Polymer	Synthesis method	Self-assembly	Application	Reference
Chol-PAMPS	Poly(2-(acrylamido)-2-methylpropanesulfonic acid) sodium salt end-capped with cholesterol	Free radical polymerization through initiator containing cholesterol moiety	Core-shell structured micelle / intermolecularly bridged “flower-type” micelles	Not Available	[12]
Chol-P(NIPAAm-*co*-DMAAm)	Cholesteryl end-capped poly(N-isopropylacrylamide-co-N,N-dimethylacrylamide)	Esterification of hydroxyl end-group of precursor polymer and cholesteryl chloroformate	Spherical, star-like, cuboid-like micelles	Temperature-sensitive drug delivery system (DDS)	[13,26]
Chol-MPC	poly[2-(methacryloyl-oxy)ethyl phosphoryl-choline] with cholesterol end-group	ATRP through macroinitiator containing cholesterol moiety	Spherical micelles	DDS carriers	[15,16]
Chol-PEG	Cholesterol terminated poly(ethylene glycol)	Commercially available	Core-shell micelles	DDS carriers	[17]
MeO-PEG-Chol	Methoxy poly(ethylene glycol)-cholesterol	Ester coupling reaction of cholesterol with end-groups of precursor polymer	Spherical micelles	DDS carriers	[18]
Chol-(L-Lactic acid)_n_	Cholesteryl end-capped oligo(L-lactic acid)	ROP initiated by the aluminum alkoxide generated in situ from triethylamine and Cholesterol	Smectic LC phase in neat state	Tissue engineering scaffolds	[23,24]
Chol-PDTC	Cholesteryl end-capped poly( 2,2-dimethyl-trimethylene carbonate)	ROP initiated by hydroxyl group in cholesterol	LC in neat state	DDS carriers	[25]
Chol-PTMC	Cholesteryl end-capped poly(trimethylene carbonate)	ROP initiated by hydroxyl group in cholesterol	LC in neat state	DDS carriers	[20]
Chol-(CL)*_n_*	Cholesterol end-capped poly(ε-caprolactone)	ROP initiated by hydroxyl group in cholesterol	LC in neat state	DDS carriers	[21]
Chol-(LG)_m+n_	Cholesterol end-capped poly(lactide-co-glycolide)	ROP initiated by hydroxyl group in cholesterol	LC in neat state	DDS carriers	[19]
Chol-PI	Cholesterol end-capped polyisoprene	Coupling of amine end-group of precursor polymer and 2-cholesteryl-2-oxo-1,3,2-dioxa-phospholane	Self-associated in cyclohexane	Not available	[22]

### 2.2. Polymers bearing cholesterol side-chains

**Figure 4:**
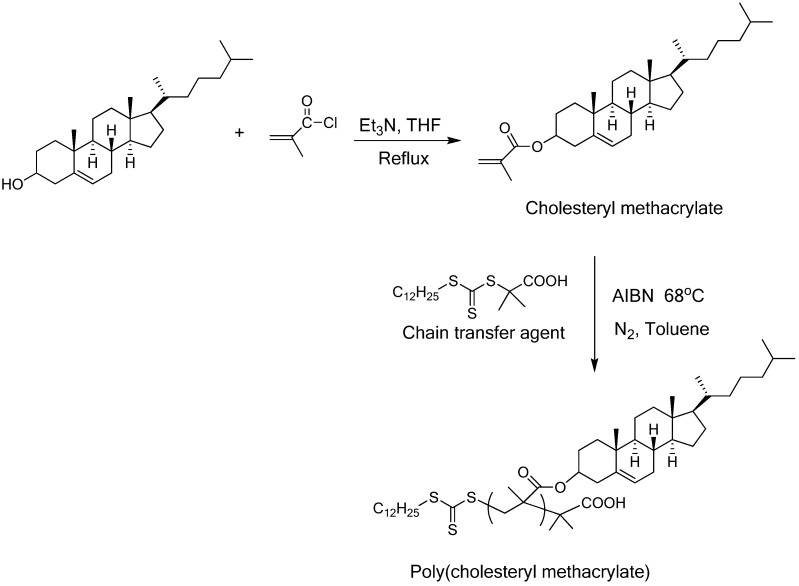
Synthesis of poly(cholesteryl methacrylate).

A variety of side-chain liquid crystalline homopolymers (SCLCPs) bearing cholesterol, have been prepared by systematically varying (1) backbone (norbornene, acrylate, methacrylate, siloxane, urethane) and (2) flexible spacers (methylene, siloxane) (Table 2) [27,28,29,30,31,32,33,34,35,36,37,38,39,40,41,42,43]. These polymers are prepared by free-radical polymerization, controlled radical polymerization including atom transfer radical polymerization (ATRP), reversible addition fragmentation and transfer (RAFT) polymerization, metal-catalyzed ring opening polymerization, graft polymerization or step-polymerization methods. An example of synthesizing polymethacrylate containing cholesterol side groups, poly(cholesteryl methacrylate) is illustrated in Figure 4. The monomer, cholesteryl methacrylate, is prepared by the esterification reaction between cholesterol and methacryloyl chloride catalyzed by triethylamine. Cholesteryl methacrylate is polymerized by RAFT method using AIBN as the initiator in the presence of a chain transfer agent, *S*-1-Dodecyl-*S*’-(α,α’-dimethyl-α’’-acetic acid)trithiocarbonate. The structure and molecular weight of the homopolymer is confirmed by nuclear magnetic resonance (NMR) and gel permeation chromatography (GPC), respectively. The poly(cholesteryl methacrylate) retains the active thioester end-group, which allows the subsequent insertion of other monomers resulting in block copolymers [44].

**Figure 5:**
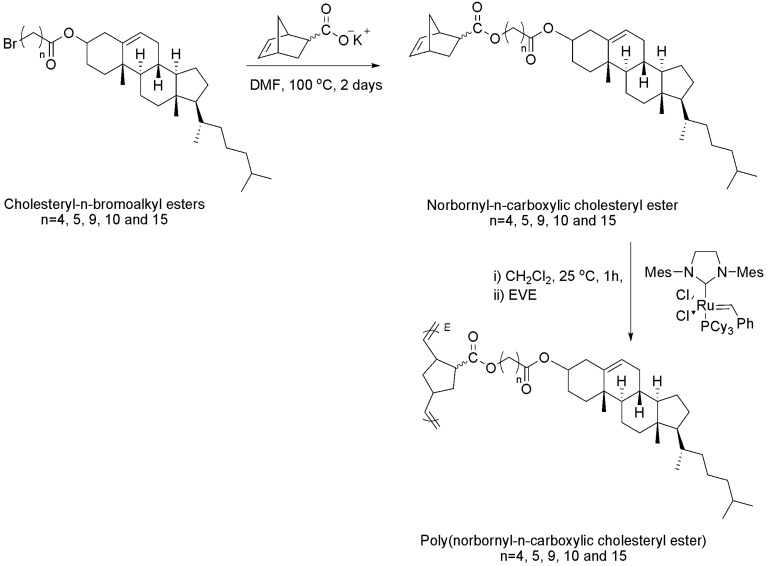
Synthesis of poly(norbornyl-*n*-carboxylic cholesteryl ester).

In another example, cholesteryl-*n*-bromoalkyl esters (Br-Chol-*n*) were first synthesized from cholesterol and *n*-alkylbromoacid chlorides, where *n* represents the number of methylene units. Monomer, norbornyl-*n*-carboxylic cholesteryl ester, (NB-Chol-*n*) was obtained from esterification of Br-Chol-*n* with norbornene carboxylate potassium salt in DMF. The monomers, (NB-Chol-*n*), were polymerized by ruthenium-catalyzed ring opening metathesis polymerization (ROMP), resulting in corresponding homopolymers, poly(norbornyl-*n*-carboxylic cholesteryl ester), (PNB-Chol-*n*) (Figure 5) [45]. The structure and molecular weight of the monomers and homopolymers were confirmed by NMR, GPC and elemental analysis. In this method, spacers can be introduced between cholesterol mesogens and polymer backbone, providing more flexibility to the mesogens for LC ordering.

**Figure 6:**
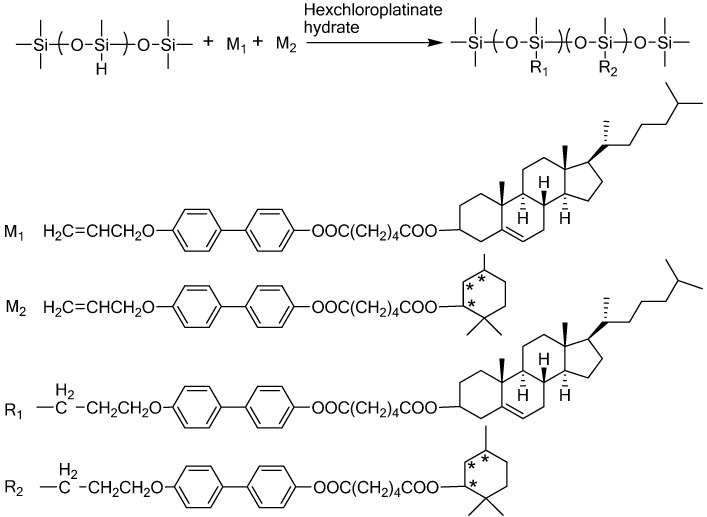
Synthesis of PMHS containing cholesterol and menthol side-groups.

Cholesterol side-chains can also be attached to polysiloxane backbones through the reaction between Si-H groups in the polymer and cholesterol bearing vinyl monomers in the presence of a Pt catalyst (Figure 6). The Si-H groups can also react with monomers containing other functional groups affording LCPs with both cholesterol mesogens and other functional groups [41].

The synthesis, self-assembly and applications of these homo-, random and block copolymers comprising cholesterol side-chains are summarized in Table 2 and Table 3.

**Table 2:** Types of homo- and block copolymers comprising cholesterol side-chains.

Homopolymers					
Abbreviation	Polymer	Synthesis method	LC observed	Application	Reference
PMHS-Chol	Poly (methylhydro-siloxane) with cholesterol side-chain	Graft polymerization	Chiral smectic	N/A	[39,41]
PNB-Chol-n	Polynorbornene bearing cholesterol with varying spacers	ROMP	Smectic	N/A	[45]
PA-Chol-n	Polyacrylate bearing cholesterol with varying spacer	Radical/photo polymerization	Cholesteric	N/A	[28,46]
PMA-Chol-n	Polymethacrylate bearing cholesterol with varying spacer	Radical polymerization	Smectic	N/A	[32,33]
**Block Copolymers**					
PMMA-Chol-*b*-PS	Poly(methylmethacrylate cholesterol-*b*-styrene)	ATRP	Smectic	N/A	[47]
PCholMA-*b*-PHEMA	Poly(cholesteryl methacrylate-*b*-2-hydroxyethyl methacrylate)	RAFT	N/A	N/A	[44]

**Table 3:** Types of random copolymers bearing cholesterol side-chains.

Polymer (Backbone + Cholesterol)	Comonomer	Synthesis method	LC observed	Application	Reference
Poly cholesteryl oleyl carbonate	Nonmesogenic spironaphthoxazine derivatives	Free radical - AIBN	Smectic / cholesteric	Photochromics such as data storage and display devices	[48]
Poly methyl-hydrosiloxane with cholesterol side-chain	Nonmesogenic chiral menthol side-chain	Graft polymerization	Cholesteric	N/A	[41]
Poly methyl-hydrosiloxane with cholesterol side-chain	Nematic benzoate side-chain	Graft polymerization	Cholesteric / blue phases	Optical storage, pyroelectric devices	[39]
Poly methylhydro-siloxane with cholesterol side-chain	Flexible cross-linking agents	Graft polymerization	Smectic / chiral smectic / cholesteric	Nonlinear optical materials, piezoelectric devices	[49,50,51]
Poly methylhydro-siloxane with cholesterol side-chain	Sulfonated cholesterol side-chain	Graft polymerization	N/A	Electro-optic displays	[38]
Poly methylhydro-siloxane with cholesterol side-chain	Cross-linking agent with sulfonic acid groups	Graft polymerization	Smectic / cholesteric	N/A	[40]
Polyacrylate with cholesterol side-chain	Alkoxybenzoic acid & smectic side-chain	Free radical - AIBN	Smectic A / nematic	Optical applications	[52]

## 3. Neat State Self-Assembly of Polymers Comprising Cholesterol

Polymers comprising cholesterol self-assemble in the neat state to produce cholesteric, smectic, nematic, and blue mesophases. Each of these mesophases has unique ordering of the cholesterol molecules resulting in tunable optical properties.

Chiral nematic or cholesteric mesophases, in which cholesterol molecules are ordered helically, reflect certain frequency ranges of incoming polarized light [53]. Due to this selective light reflection, cholesteric liquid crystals have 1D-photonic bandgap where propagation of light along the helical axis is forbidden [54]. In the edge of this stop band in which the photon group velocity approaches zero, laser action can be expected [55]. In addition to the lasing properties, control of helical pitch such that radiations are reflected with different wavelengths has been harnessed for the development of electro-optical devices, non-linear optical materials, optical data recording and storage and color recording devices [56,57,58,59,60,61,62,63,64,65].

**Figure 7:**
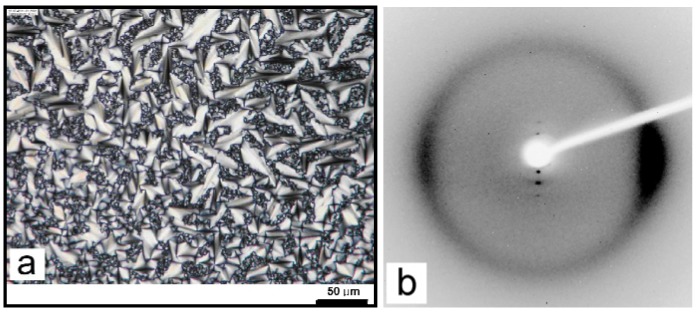
Polarizing optical microscope image (a) and 2D X-ray image (b) confirming smectic A texture of PNB-Chol-15 at room temperature.

**Figure 8:**
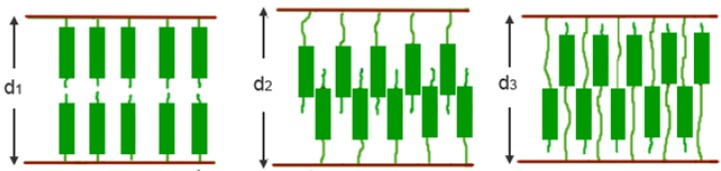
Illustration of smectic packing of side-chain liquid crystalline polymers (SCLCPs) comprising cholesterol.

Despite the presence of eight chiral centers in cholesterol, chiral nematic or cholesteric mesophases are generally not observed for homopolymers comprising cholesterol. Polynorbornenes, polysiloxanes and polymethacrylates comprising cholesterol side-chains can self-assemble to form layered smectic mesophases [41,44,45]. The polarized optical micrograph (POM) and the 2D X-ray image of polynorbornene with cholesterol arranged in smectic A mesophase are shown in Figure 7a,b, respectively [45]. The smectic or layered arrangement of cholesterol can be bilayered, partially interdigitated, or completely interdigitated single layered packing with increasing spacer length between mesogens and polymer backbones [32,45]. A cartoon depicting the formation of smectic A mesophase in polymers comprising cholesterol is shown in Figure 8.

While the formation of cholesteric mesophases have been harnessed for color information technology, lasing and optoelectronic applications [56,57,58,59,60,61,62,63,64,65], the formation of smectic A mesophase has been used to produce scaffolds for tissue engineering applications [23,24]. Stupp and coworkers have shown that the cholesterol-layered polylactide scaffolds improved fibroblast adhesion and spreading, although the specific mechanism for this observed response remains unknown. The ability of self-assembling materials to present ordered and periodic bulk structures to cells could be a useful strategy in tissue engineering [23,24].

Efforts to combine cholesterol monomers with various other mesogenic and non-mesogenic groups, chiral and achiral groups, dopants, and cross-linkers are examined. A wide variety of investigation based on methods, composition, mesophase morphology, and thermal properties of copolymers comprising cholesterol are reported [34,38,41,42,49,50,66,67,68,69,70,71,72]. Flexible cross-linkers, non-mesogenic, chiral, and photochromic groups have been used in efforts to push the commonly observed smectic LC phase to the less-ordered cholesteric phase (Table 3).

### 3.1. Non-mesogenic groups

Due to the lack of observed cholesteric phases in several LCPs bearing cholesterol, the incorporation of non-mesogenic side-chains has been investigated as an alternative route to cholesteric LCP. Two specific examples of this, (i) the incorporation of chiral non-mesogenic groups and (ii) photochromic non-mesogenic groups, have been explored.

Wang *et al.* synthesized polysiloxanes bearing randomly positioned cholesterol and menthol moieties through biphenyl and alkyl chain spacers, respectively. The addition of menthol, a non-mesogenic chiral side-chain, has been shown to push the chiral smectic A phases observed in the polysiloxane bearing cholesterol to helical Grandjean textured cholesteric phases. The authors have shown that the phase behavior of polysiloxane comprising cholesterol is strongly influenced by the incorporation of the menthyl comonomer. The helical orientation of the cholesteric phase is strengthened by the increasing amount of menthyl side-chains. Increasing the percentage of the bulky menthyl comonomer also led to an increase in glass transition temperature (T_g_) and decrease in cholesteric clearing temperature, T_cl_ [41].

In addition to the chiral non-mesogenic groups used to alter optical properties of LCPs, the influence of non-mesogenic photochromic groups has also been investigated. Hattori and Uryu incorporated non-mesogenic spironaphthoxazine derivatives into cholesteryl oleyl carbonate systems. A smectic mesophase was observed for the cholesteryl homopolymer while a cholesteric mesophase was observed for the corresponding copolymers [48].

### 3.2. Mesogenic groups

Cholesteric LC phases have also been obtained by copolymerization of monomers comprising cholesterol with monomers comprising non-chiral mesogens [39]. By incorporating non-chiral nematic monomers with monomers comprising cholesterol, nematic phases undergo transition to cholesteric due to geometric and chemical dissimilarity of the mesogens. As shown with polysiloxanes comprising cholesterol and butyl 4-[4-(2-propenyloxy)benzoxy]benzoate, the incorporation of non-chiral nematic side-chain partially disrupts the helical ordering of the chiral cholesterol mesogens. The mesophases of copolymers with varying amounts of non-chiral nematic side-chains show transition from chiral smectic to cholesteric and eventually blue phase. Blue phases, typically only observed over a small temperature range, are cubic lattices with no positional long-range order. Blue phases can be characterized by higher order Bragg reflections typical for cubic lattices in X-ray diffraction and blue planar textures observed in POM. The cholesteric phase for these copolymers were observed for a temperature range of 70°C followed by blue phase for a range of 20 °C [39].

### 3.3. Elastomers

Zhang and coworkers have used various cross-linkers to alter the properties and mesophases of cholesteric LCPs [49,50,51]. They first showed that the use of flexible cross-linkers (less than 20% by weight) do not disturb the LC packing [51]. The use of a chiral isosorbide cross-linker, with increase of concentration from 0 – 20%, altered the LC phase from smectic to chiral smectic C to cholesteric. The authors proposed that the chirality of the cross-linker was the promoting factor in the alteration of observed mesophases and optical properties.

### 3.4. Dopants and ionic groups

Ionic groups, such as sulfonic acid, carboxylic acid and sulfonate moieties, have been added to the backbone and side-chains in order to tailor the LC properties of cholesterol side-chain polymers. Zhang *et al.* have showed that the addition of sulfonate groups to cholesteryl side-chains did not influence the T_g_ even though it is observed in traditional ionic polymers.[38] Copolymers containing 0.09 – 0.70 wt% K^+^ showed only a single cholesteric mesophase while increasing the percentage to 1.05% led to the incorporation of a smectic-cholesteric transition at 117.9 °C.

By adding ionic groups to chemical cross-linking agents, Zhang *et al.* were also able to investigate the influence of ionic character on LC elastomers. It was determined that increasing the amount of sulfonic acid groups from 0-1% causes a decrease in the LC range for the cholesteryl siloxane polymers with a loss of LC character for percentages higher than 1% [40].

**Figure 9:**
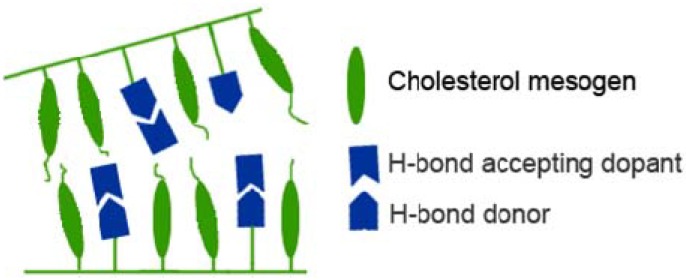
Schematic illustration of LC polymer blends with hydrogen accepting dopants.

Mallia *et al.* synthesized a series of glassy LC oligomers containing cholesterol mesogens; low molar mass dopants were added to these polymers to obtain cholesteric mesophases [52,73,74,75,76,77,78,79,80,81,82,83]. This transition to cholesteric has been shown with side-chain polymers containing smectic, cholesteryl and benzoic acid side-chains. Three pyridine-containing dopants were used to disrupt the smectic-layered structure in efforts to form a helical structure. A schematic illustration of hydrogen-bonded dopants disrupting smectic layers of cholesterol side-chain polymers is shown in Figure 9.

Our group has synthesized polynorbornenes bearing randomly distributed cholesterol and carboxylic acid groups. The amount of carboxylic group has been varied from 10 – 50 wt %. The T_g_ and T_cl_ can be tailored by the incorporation of carboxylic acid groups, unlike previous reports [38]. Analysis of resultant mesophases in polymers comprising cholesterol and carboxylic acids by POM and X-ray is currently under progress (Briand, V.; Ahn, S. K.; Kasi, R. unpublished data).

In summary, neat or melt-state ordering of polymers comprising cholesterol has been used to investigate fundamental phase behavior of the LC states. These LCPs can form smectic A or C, nematic, cholesteric or blue mesophases. The thermal properties, T_g_ and T_cl_, of these LCPs can be tailored by the choice of the backbone, comonomer, dopants and degree of cross-linking. The formation of mesophases is harnessed for specific applications. For example, cholesteric and blue mesophases are used in optoelectronic, color information technology and lasing applications. Polymers comprising cholesterol in (1) smectic A mesophase are used for tissue engineering applications and (2) smectic C are used for generating artificial muscles and actuators.

## 4. Solution State Self-Assembly of Polymers Comprising Cholesterol

Over the past few decades, solution self-assembly of hydrophobically modified (HM) water-soluble polymers has been a subject of great interest [84,85,86,87,88,89,90,91,92]. The association involves both intra- and intermolecular interactions of the hydrophobes attached as a pendant group or at the terminal end of a water-soluble polymer. It is usually observed that intramolecular associations occur at low concentrations, which leads to the formation of micelles, while at higher concentrations, there is a strong propensity of intermolecular association resulting in gelation and precipitation [84,85,86,87,88,89,90,91,92]. Selection of hydrophobic moiety for HM water soluble polymer is a crucial and challenging task because of a wide variety of constraints offered from synthetic procedures and opportunities for potential application areas. Cholesterol is chosen as a preferred hydrophobe, not only because it is biodegradable and biocompatible but also high thermodynamic affinity for the cell membrane and its ability to change the membrane’s permeability [93,94].

According to recent evidence, cholesterol is important in the stability of lipid rafts, important sites for membrane receptors [95]. Thus, cholesterol delivery to cells could be important in signal transduction, cell adhesion with its biological consequences, and cell migration. In addition, mesogenic character and ability to form liquid crystalline phases could provide a driving force for the self-assembly of the macromolecules [96,97]. A striking feature seen that a “small number” of cholesterol units attached as pendant or end group can induce formation of structures of well-defined morphology in water as a result of specific interactions among the cholesterol moieties.

### 4.1. Micelles formed by polymers comprising cholesterol

**Figure 10:**
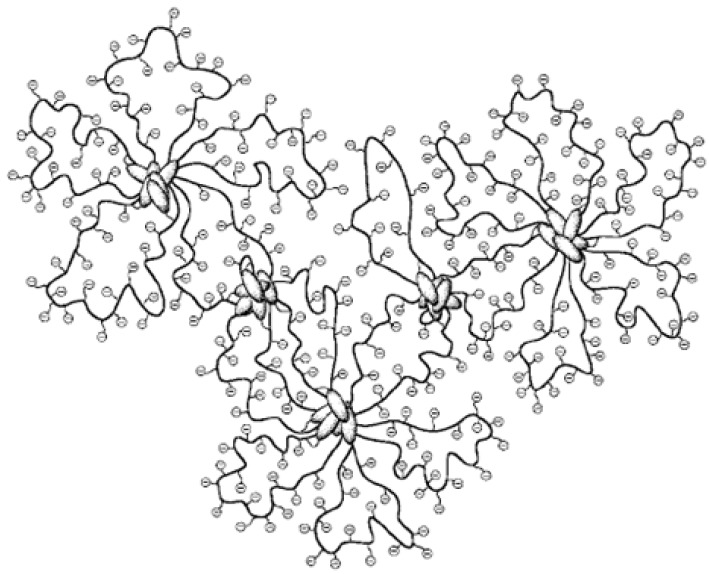
Illustration of intermolecularly bridged flower-type micelles self-assembled by random copolymers of sodium 2-(acrylamido)-2-methylpropanesulfonate (AMPS) and cholesteryl 6-methacryloyloxyhexanoate (Chol-C-5-MA). (Reprinted with permission from Reference [84]).

Two types of polyelectrolytes carrying cholesterol (Chol) pendants were synthesized by the copolymerization of sodium 2-(acrylamido)-2-methylpropanesulfonate (AMPS) with cholesteryl methacrylate (CholMA) or cholesteryl 6-methacryloyloxyhexanoate (Chol-C-5-MA). The CholMA and Chol-C-5-MA units in the copolymers range from 0.5 to 1.0 and 0.5 to 10 mol%, respectively. Using fluorescence, static light scattering (SLS) and quasielastic light scattering, it was found that the pentamethylene spacer can facilitate the interpolymer association of cholesterol pendants. An intermolecularly bridged “flower-type” micelle model for the aggregates of the Chol-C-5-MA copolymers have been proposed (Figure 10) [84].

The formation of flower-type micelles were also observed in random copolymers of AMPS and Chol-C-5-MA in 0.1 M NaCl aqueous solution [98]. In another related study, it was observed that concentrations higher than 5.0 g/L, poly(2-(acrylamido)-2-methylpropanesulfonic acid) sodium salt end-capped with cholesterol moiety (Chol-PAMPS) forms aggregates with size too large for a single spherical micelle. The presence of polymer chains with cholesterol groups at both ends result in the overlap of cholesterol such that spherical micelles are bridged by these polymer chains [12]. The binding of bile salt with cholesterol containing polymers in aqueous solution has also been studied. A HM polysulfonate bearing a small mole percent (5 mol %) of cholesteryl moieties forms intermolecularly bridged flower micelle in the aqueous solution which can be disrupted upon the addition of sodium cholate (SC) through interactions between the cholesterol groups in the polymers and SC [99].

**Figure 11:**
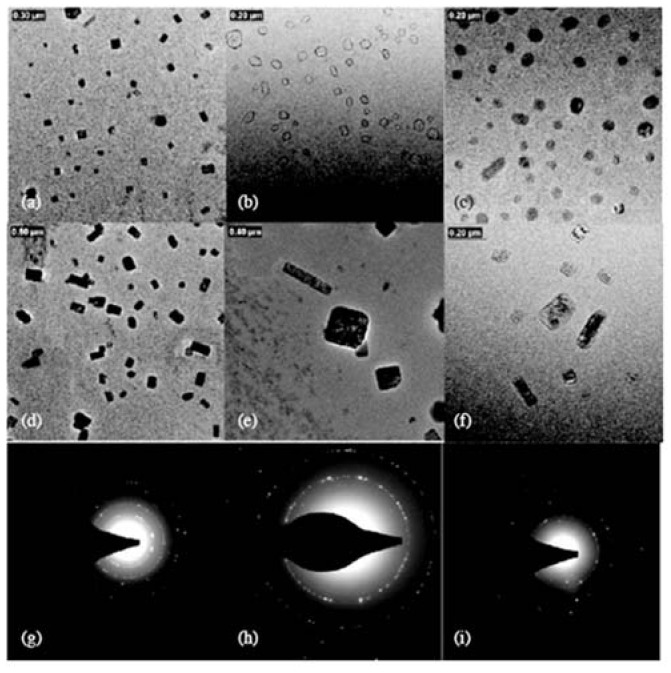
Nanoparticles self-dried from the micelle solutions of poly(*N*-isopropylacrylamide-co-*N*-hydroxymethylacrylamide) bearing cholesterol side-chains with a polymer concentration of 0.1% (a, d, e); 0.3% (b, f) and 0.6% (c). Electron diffraction patterns of particles of d (g, h) and c (i). (Reprinted with permission from Reference [13]).

Amphiphilic copolymers containing poly(*N*-isopropylacrylamide) (NIPAmm) and cholesterol have been prepared. These copolymers take advantages of both the temperature sensitivity of NIPAmm and the targeting capability of cholesterol to the receptors on the cell membranes. Both main-chain (cholesterol end-capped) and side-chain types of poly(*N*-isopropylacrylamide-co-*N,N*-dimethylacrylamide) have been synthesized and their self-assembly in aqueous solution have been studied. These amphiphilic polymers self-assemble to form micelles with different morphologies including spherical, star-like, cubic or cuboid-like shapes upon changing the polymer concentration (Figure 11) [13,14]. The rigidity of the cholesterol molecule and its propensity for forming layered structures accounts for the novel morphologies. These self-assembled structures were used to encapsulate drugs cyclosporin A (CyA) and indomethacin (IND) and subsequent drug release behaviors were studied [100]. The cholesteryl modified polymers yielded a higher encapsulation efficiency for drugs compared to control polymers; better entrapment was observed for IND compared to CyA. IND release from the nanoparticles was responsive to temperature changes, being faster at a temperature around the lower critical solution temperature (LCST) than below the LCST.

Random copolymers of *N*-isopropylacrylamide (NIPAmm) and cholesteryl acrylate have been prepared. By changing the ratio of NIPAmm to cholesteryl acrylate, the lower critical solution temperature (LCST) and the amphiphilic properties of the copolymers can be tuned [101]. Cholesterol-grafted poly(*N*-isopropylacrylamide-co-*N,N*-dimethylacrylamide-co-undecenoic acid) was synthesized and folate or folic acid was attached to the hydrophilic segment. Folate and folic acid are capable of targeting the folate receptors in over-expressing cancer cells. As a result, micelles fabricated from this copolymer can be used to carry anti-cancer drugs such as doxorubicin [102]. The drug can be released by changing the pH of the medium. The drug delivered by the cholesterol- and folate-conjugated cholesteryl polymers entered nucleus more rapidly than the control unmodified polymers. More importantly, the folate-conjugated cholesteryl polymers yield a greater cellular uptake because of the folate-receptor-mediated endocytosis process. These multifunctional polymer core/shell nanoparticles may make a promising carrier to target drugs to cancer cells and release the drug molecules to the cytoplasm inside the cells [101,102].

**Figure 12:**
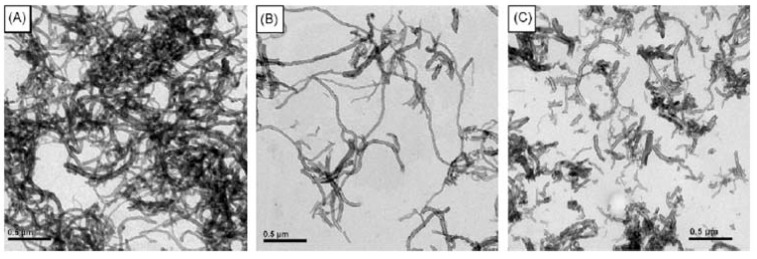
TEM images for (A) the pristine CNTs, (B) the CPMPC coated CNTs, (C) chemically oxidized CNTs-COOH. (Reprinted with permission from Reference [103]).

Xu *et al.* prepared poly[2-(methacryloyloxy)ethyl phosphorylcholine] with cholesterol end-groups (CPMPC). These amphiphilic polymers self-assemble in aqueous solution to form spherical micelles which could be used to deliver hydrophobic drugs [16]. Furthermore, this copolymer can non-covalently bind with the surfaces of carbon nanotubes (CNTs) [103]. While the pristine CNTs aggregates as large bundles, CNTs bound with CPMPC were well-dispersed in aqueous solution (Figure 12). This is attributed to the association of cholesterol moieties of the amphiphilic polymers with the CNTs via hydrophobic-hydrophobic interactions and hydrophilic segments of the copolymers disperse CNTs in water. The solubility of CNTs in water can be increased using carboxylation chemistry which will chop the CNTs fibers to short pieces while CPMPC solubilization will keep the CNTs intact.

Poly(amidoamine)-cholesterol conjugated polymers have been prepared by attaching cholesterol moieties to the poly(amidoamine) (PAA) backbones via disulfide linkage. These polymers self-assemble to form nanoparticles with cholesterol forming hydrophobic core and PAA forming the corona. These nanoparticles show a redox-sensitivity, since the disulfide linkage will be stable in the blood but can be cleaved inside the cells, which makes them good candidates for biomedical applications [104].

Cholesterol can be introduced into polyethylene glycol (PEG) by end-group functionalization. The study of commercially available cholesterol terminated PEG (Chol-PEG) showed that the PEG block forms the biocompatible micelle coronas and the cholesterol block formed the hydrophobic micelle cores [17,18]. Other examples of PEG functionalized with cholesterol include peptide-PEG-cholesterol conjugates, copolymers of methoxy poly(ethylene glycol) (mPEG)-acrylate, 2-hydroxyethyl methacrylate (HEMA)–cholesterol conjugates and HEMA, and PEGylated poly[(*N*-methyl-dietheneamine sebacate)-co-[(cholesteryl oxocarbonylamido ethyl) methyl bis(ethylene) ammonium bromide] sebacate] (PEGylated P(MDS-co-CES)) [106,107,108]. An increased cholesterol grafting degree led to greater gene expression level, which the authors attribute to more stable core–shell structure of the micelles. Specifically, these micelles induced high gene transfection results compared to control polymers. These biomimetic micelles and mixed micelles have been tailored with specific binding abilities, release kinetics, and cytotoxicity that can be used for the delivery of hydrophobic drugs, plasmids, and nucleic acids [17,18,105,106,107].

Comb-like linear polymers have been prepared with different hydrophilic backbones and cholesterol side groups as hydrophobic components. These comb-like polymers self-assemble to form spherical micelles in aqueous solution. Thompson *et al.* attached cholesterol moieties onto poly(allylamine) (PAA) backbone as side-chains [108]. These polymers self-assemble to form dense nanoparticles in aqueous solution and furthermore they were transformed into nanostructures including nanoparticles with cholesterol-enriched dense core coated by PAA, or elongated or lamellar nanoparticles in the presence of free cholesterols. Other examples of comb-like polymers that form micelles include N-cholesterol succinyl *O*-carboxymethyl chitosan (CCMC). The morphology and size of the micelles have been studied with TEM and light scattering. The drug release behaviors of the micelles have also been studied by using a model drug [109].

Akiyoshi *et al.* studied the self-organization of cholesteryl-bearing pullulans (CHP) in water. These polymers form stable, monodisperse nanoparticles by intermolecular self-aggregation in dilute aqueous solution and gels in semi dilute solution. Irrespective of the molecular weight of the parent pullulan and degree of substitution (DS) of the cholesteryl moiety, CHPs provided unimodal and monodisperse aggregates in water. The size of the aggregate decreased with an increase in the DS of the cholesteryl moiety. Additionally, these hydrophobic aggregate are unperturbed by the collapse or expansion of the main chain. This unique property can be used to encapsulate protein folding aids or thermal stabilizer of enzymes and release them by using specific triggers [93,110,111,112].

Our group has prepared micelles from amphiphilic diblock copolymer poly(cholesteryl methacrylate)-*b*-poly(2-hydroxyethyl methacrylate), PCMA-PHEMA (Zhou, Y.; Kasi, R. unpublished data). In aqueous solution, the PCMA-PHEMA self-assembles to form spherical micelles with PCMA as the hydrophobic core and PHEMA as the hydrophilic corona. These micelles may be used to encapsulate and deliver therapeutics, contrast agents and hydrophobic drugs.

### 4.2. Physical gels formed by self-assembled cholesterol containing polymers

**Figure 13:**
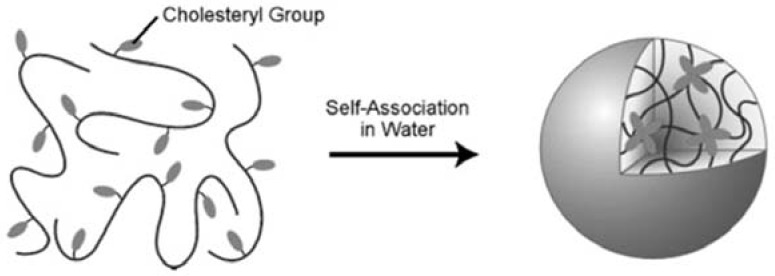
Schematic illustration of the formation of a nanogel by cholesterol-bearing pullulan molecules through the self-association of cholesteryl groups in water. (Reprinted with permission from Reference [114]).

Self-assembled hydrogels have been prepared with pullulan bearing cholesteryl groups (CHP). The side-chain cholesterol groups function as physical cross-linkers that drive the formation of hydrogel in dilute aqueous solutions (Figure 13). Polymerizable methacrylate side groups were also introduced into CHP, resulting in CHPMA. This product was subsequently cross-linked via the copolymerization with 2-methacryloyloxyethyl phosphorylcholine (MPC). Thus, a dual cross-linking nanogel system was achieved containing both the physical cross-linking of cholesterol groups and the chemical cross-linking from MPC. While the physical cross-linking of cholesterols is important for the interaction of protein with nanogels, the MPC network can be used to trap the protein, making the cross-linked CHPMA nanogel a good candidate for delivery systems [113]. The complexed protein in the gel can be released by interaction of β-cyclodextrin (β-CD), which dissolving the physical crosslinks within the CHP nanogel. The nanogels can be used as carrier systems in biotechnology. Additionally, these nanogels can also be used as templates for calcium phosphate mineralization. These nanohybrids may offer a new type of pH-sensitive hybrid drug carriers [114].

Hydrophobic modification of polyelectrolyte, poly(L-lysine) (PLL), has been accomplished by attaching cholesterol moieties. The modified PLL with cholesterol, CHPLL, forms hydrogel along with unique polypeptide conformations in aqueous solutions [112]. Results obtained from static light scattering showed an increase in particle size (<R_G_>) and aggregation number (N_agg_) with an increase in the degree of substitution of cholesterol in CHPLL (<R_G_>: 16 – 22 nm, N_agg_: 1.3 – 4.2). Using circular dichroism, the authors have shown that these CHPLLs form α-helical structures at lower pH values unlike parent PLL, probably caused by the association of cholesteryl groups. The α-helical content of CHPLLs can be decreased upon the addition of β-cyclodextrin which forms inclusion complex with cholesterol. This is an evidence of the association of cholesterol groups affecting the conformation of PLL. These hydrogels can be used as drug delivery carriers and artificial molecular chaperons for biomedical applications [112].

Recently, star-shaped cholesterol-substituted 8-arm poly(ethylene glycol)-block-poly(L-lactide) (8-arm PEG-*b*-PLLA-cholesterol) have been prepared. The number of cholesterol units at PLLA chain ends is 2.3 – 7.8. A sol-gel transition was observed around 34 °C for this polymer in aqueous solution. It was proposed that this sol-gel transition was induced by the inter-micellar association or overlapping of cholesterol groups. The hydrogel formed from this copolymer is a promising injectable tissue-engineering scaffold [91]. In another study, star-shaped PEG with cholesterol end-group self-assembles with star-shaped PEG with β-cyclodextrin end-groups (β-CD) in aqueous solution to form hydrogels. The formation of hydrogel could be attributed to the formation of cholesterol/β-CD inclusion complex [115,116]. When the polymers are dissolved in water, the hydrophobic cholesterol moieties can be inserted inside the CD, providing intermolecular physical cross-linking that results in gelation [117].

LC hydrogels have been prepared from copolymers composed of 5-acryloyloxypentyl cholesterate (Ch5A) and acrylic acid (AA). Copolymers with Ch5A molar fraction of 0.05 or higher form hydrogels in water. The hydrogel formation is attributed to the stacking of cholesterol groups supported by the hydrogen bonding of AA with water. From the polarized optical microscopy and X-ray diffraction studies, bilayered smectic mesomorphic phases are observed in the prepared hydrogels, which is similar to that observed in the dry polymer [118].

### 4.3. Solution properties of polymers comprising cholesterol in an organic solvent

Polymers comprising cholesterols form lyotropic LC phases due to the association of cholesterol in a solvent selective to either the polymer backbone or the cholesterol molecules [119]. For example, the self-assembly of polyisoprenes bearing phosphatidylcholine analogues chemically connected with cholesterols in cyclohexane have been studied. The zwitterionic end-groups leads to the formation of aggregates in cyclohexane, while LC packing of cholesterol results in higher aggregation number. In another study, poly(cholesteryl 6-(methacryloyloxy)hexanoate) were prepared and a small amount of (1-pyrenylmethyl) 6-(methacryloyloxy)hexanoate (Py-C5-MA) were added as a fluorescence probe via copolymerization. Wide angle X-ray and fluorescence studies showed that the cholesterol moieties in the copolymers forms stacks in *n*-hexane [119]. These stacks of cholesterol groups of the copolymers in *n*-hexane have been utilized as microdomains to confine zinc(II) tetraphenylporphyrin (ZnTPP), an important agent in the photosynthetic process. Isolation of ZnTPP will afford an easy route to study photophysical and photochemical behaviors of ZnTPP in the photosynthetic process [120].

In summary, solution state ordering of polymers comprising cholesterol has been used to investigate fundamental phase behavior. These LCPs form micelles, nanoparticles, hydro-, organo- or lyotropic gels. The size, mechanical and thermal properties of these polymers are designed for encapsulation, storage, release and targeted delivery of therapeutics, gel-based scaffolding materials, templates for biomineralization, and nanocontainers for photosynthetic donors and receptor.

## 5. Summary: Current and Future Directions

In this article, concurrent developments in synthesis and structure-property relationship of polymers comprising cholesterol have been reviewed. These fundamental studies are essential to discover and understand various aspects of hierarchical ordering of cholesterol and the physical properties of the mesophases. These investigations are critical to tailor LCP properties for applications in targeted delivery of cancer drugs, genes, and plasmids, MRI contrast agents, scaffolds that direct growth of tissues, optoelectronics, lasing materials and information technology. Holistic modeling and experimental investigations on the interaction of these polymers with cell lines are significant to the understanding of delivery mechanisms by which these polymers function as therapeutics. Detailed investigations of responsive characteristics of different mesophases in conjunction with design of devices and modeling will be critical for the development of these polymers in display technology.

Future research efforts will be targeted towards polymers comprising cholesterol and other functional groups. These self-assembled materials will be used to optimize critical parameters to discover new mesophases at multiple length-scales in the melt and in solutions. Nanoscopic assemblies (5 – 10 nm) of these LCPs will be exploited to design new materials for renewable energy applications. Production of these assemblies with long-range order (nm-μm) will enable the transport of charge carriers to appropriate electrodes, facilitating the design of efficient solar- and fuel cells. Development of combinatorial evaluation methods to investigate physical, chemical, biological, thermal, optical, rheological and responsive properties of these new polymer libraries will be equally important. In summary, multiple length-scale self-assembly of these functional polymers will be harnessed to design the next generation of delivery vehicles, medical devices, photovoltaics, optoelectronics, thermoelectric and fuel cell devices.
